# Analysis of the gut microbiome in sled dogs reveals glucosamine- and activity-related effects on gut microbial composition

**DOI:** 10.3389/fvets.2024.1272711

**Published:** 2024-02-07

**Authors:** Dong Wang, William A. Russel, Kaitlyn M. Macdonald, Valerie M. De Leon, Ahmet Ay, Kenneth D. Belanger

**Affiliations:** ^1^Department of Computer Science, Colgate University, Hamilton, NY, United States; ^2^Department of Mathematics, Colgate University, Hamilton, NY, United States; ^3^Department of Biology, Colgate University, Hamilton, NY, United States

**Keywords:** microbiome, glucosamine, cosequin, canine health, gut health, microbial diversity

## Abstract

The composition of the microbiome influences many aspects of physiology and health, and can be altered by environmental factors, including diet and activity. Glucosamine is a dietary supplement often administered to address arthritic symptoms in humans, dogs, and other mammals. To investigate how gut microbial composition varies with glucosamine supplementation, we performed 16S rRNA sequence analysis of fecal samples from 24 Alaskan and Inuit huskies and used mixed effects models to investigate associations with activity, age, and additional factors. Glucosamine ingestion, age, activity, sex, and diet were correlated with differences in alpha-diversity, with diversity decreasing in dogs consuming glucosamine. Beta-diversity analysis revealed clustering of dogs based on glucosamine supplementation status. Glucosamine supplementation and exercise-related activity were associated with greater inter-individual pairwise distances. At the family level, *Lactobacillaceae* and *Anaerovoracaceae* relative abundances were lower in supplemented dogs when activity was accounted for. At the genus level, *Eubacterium* [*brachy*], *Sellimonus*, *Parvibacter*, and an unclassified genus belonging to the same family as *Parvibacter* (*Eggerthellaceae*) all were lower in supplemented dogs, but only significantly so post-activity. Our findings suggest that glucosamine supplementation alters microbiome composition in sled dogs, particularly in the context of exercise-related activity.

## Introduction

More than 30 trillion microorganisms inhabit the human body (1). A significant portion of these microorganisms reside in the digestive tract, collectively containing more than 3 million genes that comprise the gut microbiome (2), which is the largest and most diverse microbiome in the human body (3). In its coevolution with humans, the functions of the microbiome have become intertwined with human physiology (4, 5). As such, the gut microbiota has been implicated in important physiological functions in the human body, including but not limited to gut barrier stabilization, development of host immune response, endocrine function, and neurological signaling and development (6–9). As a result, the gut microbiome plays an important role in health and disease (10), emerging as an intriguing area of study.

Animal models have proven useful for understanding factors that influence and are influenced by the gut microbiome (11). For example, murine models have been utilized to establish causal links between the gut microbiome and disease (12, 13). Despite this promise, our ability to extrapolate findings from mice and other rodents is limited since only 4% of microbial identity is shared between mice and humans (14). Additionally, evolution has facilitated a notable divide between the biological processes of humans and mice, which further limits the utility of studying the gut microbiota of mice to inform our understanding of human processes (15).

Given the co-evolution between humans and dogs, canine models have pertinent applications for the study of gut microbiota. Dogs share a similar diet to humans, possessing omnivorous metabolism and being able to digest, absorb, and metabolize dietary carbohydrates (16, 17). Moreover, dogs, in contrast to mice, share similar microbiome composition to humans as they have historically shared a similar environment and lifestyle (18). Working sled dogs are frequently used as models for understanding the effects of exercise on physiology and health (19). Sled dogs and other intensely exercising dogs frequently experience exercise-induced gastric disease (EIGD), characterized by gastric lesions and increased intestinal permeability (20–22). Recent work has provided evidence that exercise in racing sled dogs alters the microbiome, resulting in an increase in beneficial microbes and a concomitant decrease in dysbiosis-associated bacteria and supplementation of sled dogs with a targeted symbiotic altered the microbiome and decreased diarrhea, potentially due to a transient increase in Lactobacillaceae and Streptococcaceae and decreases in Clostridiaceae (23, 24). However, the ability of dietary supplements to alter the microbiome is unclear.

In the United States, inflammatory arthritis is a leading cause of disability among adults (25, 26) and dogs (27, 28). Two of the most commonly taken supplements for joint inflammation in humans and dogs are the sulfated carbohydrates chondroitin and glucosamine (29–31). Despite their long history as a treatment for joint pain (32), there is controversy regarding the benefits of glucosamine and chondroitin for joint health, leading some experts to recommend these supplements as an adjunctive treatment (33, 34), and some experts not to recommend supplementation at all (35, 36) based on evidence that glucosamine and chondroitin do not have beneficial effects (37, 38) or may be detrimental (39). Overall, glucosamine and chondroitin exhibit poor absorption in the mammalian intestine (40, 41) and are largely consumed by the gut microflora before they can be absorbed (41). These findings have led to speculation that glucosamine and chondroitin may be indirectly involved in the production of anti-inflammatory compounds through gut bacterial pathways (42, 43). While few studies have examined the exclusive effects of glucosamine and/or chondroitin supplementation on the gut microflora in any mammal, two studies in humans have suggested that glucosamine intake has effects on the relative abundance of specific microbial taxa (44, 45).

Given the intriguing evidence that glucosamine supplementation may affect joint inflammation through the gut microbiome, we sought to examine the effects of glucosamine supplementation on gut microbial composition of sled dogs in this study. We also considered age, sex, breed, diet, health status, and exercise-related activity as potential influences on fecal microbiome composition, since all these factors have previously been suggested to affect the gut microbiome in dogs (46–50). In this retrospective study, we investigate the links between microbiome composition, glucosamine supplementation, and physical activity.

## Methods

### Data collection and processing

Twenty-four adult sled dogs (11 males; 13 females) aged between 2 and 12 years old were examined in this study. One pre-activity fecal sample was collected from each dog on January 27, 2022, and a post-activity sample was collected on January 31, 2022. Samples were shipped on dry ice and stored frozen at -20°C. Between sample collections, dogs were run 2–3 times per day in teams of four (or six for dogs 10 years old and older) for 3–7 miles per run. We refer to this higher than normal exercise for the sprint sled dogs as “activity.” Variables used in the multivariate and mixed methods analyses were: age in years, sex, breed (pure-bred Inuit husky versus mixed-breed Alaskan husky), weight, diet (Annamaet Plus kibble versus Annamaet Option canned), supplemental diet (eggs, liver, and/or Caribou Creek supplement), history of recent injury or illness, and glucosamine supplementation. For this retrospective study, glucosamine supplementation includes daily intake of either a commercial glucosamine supplement with ascorbic acid or glucosamine as a component of cosequin. Information was also collected on deworming and anti-inflammatory drug intake but not included in analyses as all dogs had recently been treated with Ivermectin and only two dogs received carprofen as an anti-inflammatory.

DNA was extracted using the DNeasy PowerSoil Pro kit (Qiagen Cat# 47014^1^) according to manufacturer’s instructions using up to 250 mg of fecal sample per extraction. Homogenization was performed using two 30-s pulses on a Beadbeater (BioSpec, Inc.) at 4°C. Microbial DNA extraction was confirmed by PCR amplification of the V4 variable region of 16S rDNA using primers 515F-Y (51) and 806R (52) according to Earth Microbiome Project (EMP) protocols (53).^2^ DNA sequencing of the 16S V4 variable region was performed by the Cornell University Biotechnology Resource Center using Illumina MiSeq and 515F-Y and 806R primers. 16S rDNA concentration after initial amplification and the number of sequencing reads in each sample are provided in Supplementary Table S1. Indexing was performed by diluting each PCR product to 2 ng/ul and using 10 ng per indexing reaction with a 5 uM concentration of i7 and i5 indexing primers (Illumina, Inc. San Diego, CA, United States).

Qiime 2 version 2022.11 was used to process the sequence reads (54). After demultiplexing, Dada2 was used to trim and denoise the sequences (55). We then obtained the feature table and representative sequences. Taxonomy was assigned using the SILVA 16S rRNA database (56). Rarefaction was done with a sampling depth of 58,667 based on the minimum read length.

### Alpha diversity analysis

Linear mixed effects models were used to investigate the relationship between selected factors and sample alpha diversity. Age, sex, breed, diet, history of illness or injury, pre- or post-activity, and glucosamine supplementation were selected as variables of interest. Alpha diversity for each sample was calculated using Shannon’s Diversity Index (57) and Faith’s Phylogenetic Diversity (58). The variables of interest were set as independent variables and the alpha diversity metric as the response variable in our models to test for significant associations.

After applying linear mixed effects models on all 48 data points, the samples were split into subgroups depending on each dog’s activity level and glucosamine treatment, and alpha-diversity calculations using Shannon’s Diversity Index and Faith’s Phylogenetic Diversity were repeated to test for subgroup associations. For the pre-activity (Pre-Ac) and post-activity (Post-Ac) subgroups, linear regression models were used. For yes-glucosamine (YG) and no-glucosamine (NG) subgroups, linear mixed effects models for repeated measures were used since each dog had both a Pre-Ac and a Post-Ac sample. For the latter two subgroups diet, breed, and injury variables were eliminated due to lack of variance. Type III F-tests were then performed in all linear models to test for significance.

### Beta diversity analysis

Beta diversity was computed using the Bray-Curtis distance metric to investigate across-sample microbial diversity (59). Microbial differences between samples were visualized in principal coordinate analysis (PCoA) using the QIIME 2 plugin Emperor (60). We then applied statistical tests to analyze beta diversity differences. For each dog, the Bray-Curtis distance was computed from its pre-activity to post-activity sample. The Wilcoxon rank sum test (61) was employed to test for differences between these distances based on glucosamine groups.

In addition to variation within the same subject over time, microbial diversity was investigated between subjects. Samples were partitioned into groups based on date of sample collection into pre-activity (Pre-Ac) and post-activity (Post-Ac) groups for which glucosamine was the variable of interest. Samples were also separated into yes-glucosamine (YG) and no-glucosamine (NG) groups. For the YG and NG groups, activity was the variable of interest. Pairwise Bray-Curtis distance was calculated in each group. Based on the type of pairs (pre-post, pre-pre, post-post or YG-NG, YG-YG, NG-NG), the Kruskal-Wallis test (62) and pairwise Wilcoxon rank sum test (61) were applied for comparison.

For both beta diversity analyses, the distances were plotted in box plots grouped by the variable of interest using ggplot2 (63) in R (64).

### Differential abundance

Differential abundance testing was carried out on the feature table collapsed on the family and genus levels. Taxonomic bar plots of relative abundances of prevalent families and genera were first generated in QIIME 2 to visualize the microbial compositions and identify important taxa. Samples were then separated by both activity and glucosamine into four subgroups (Pre-Ac/YG; Pre-Ac/NG; Post-Ac/YG; Post-Ac/NG). For each pair of subgroups, Analysis of Compositions of Microbiomes (ANCOM) was applied to identify which taxa are differentially abundant on both the family and genus levels (65). For the identified differential taxa, relative abundance levels in the four subgroups were plotted in box plots using ggplot2 (63) in R (66).

## Results

### 16S rDNA amplification and sequencing

To investigate the presence and relative abundance of microbial taxa present in fecal samples from each sled dog before and after the defined period of activity, we isolated DNA from each sample, amplified the V4 region of rDNA, and performed DNA sequencing using Illumina MiSeq. We obtained 8,408,194 high-quality 16S rRNA V4 region sequences over the 48 samples sequenced, providing an average of 175,170.7 (±19,264.7 S.D.) reads per sample. The minimum trimmed sequence length used in analysis was 251 nucleotides.

### Alpha-diversity comparison with demographic factors

**Figure 1:**
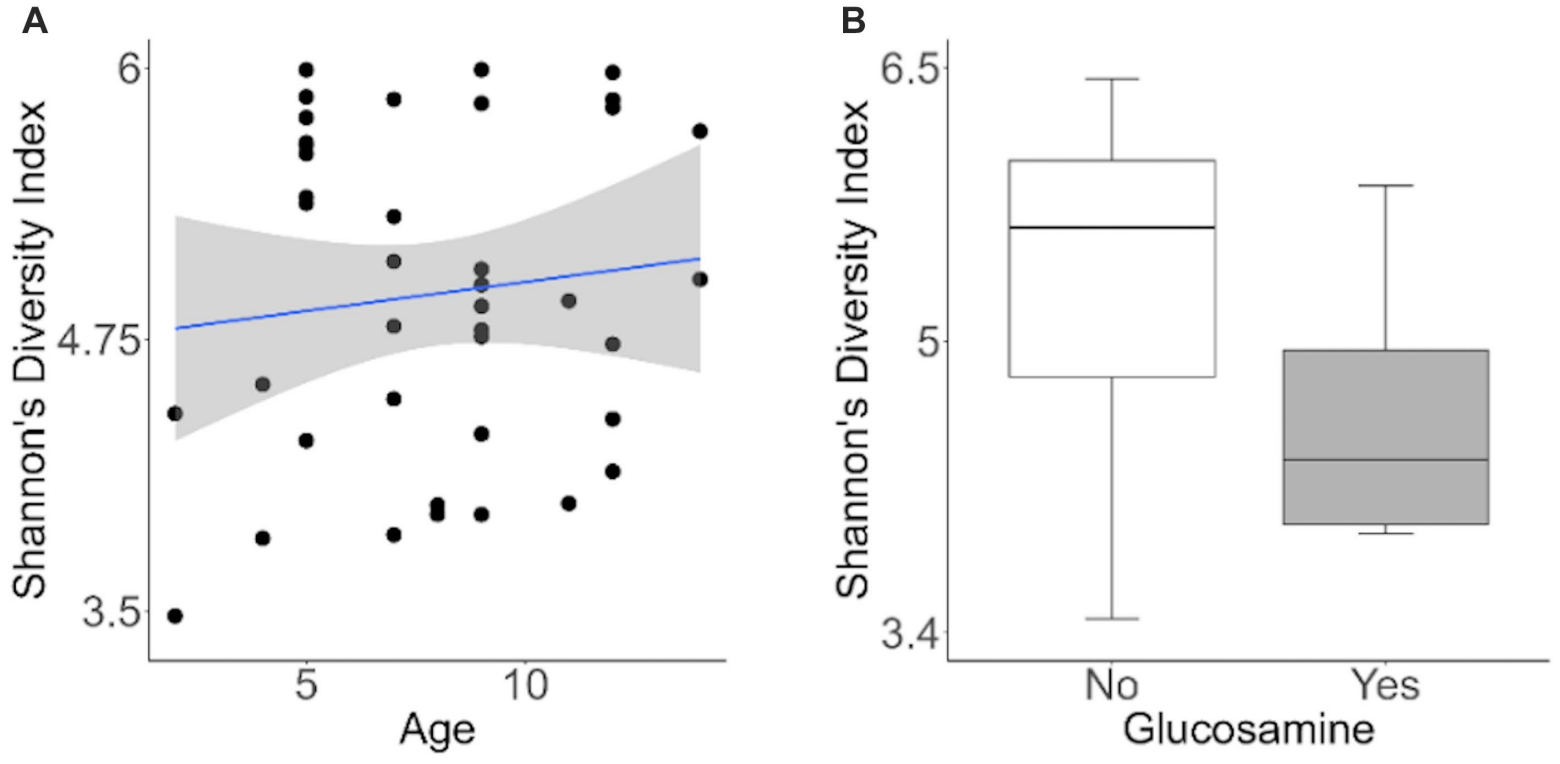
Alpha-diversity decreases with glucosamine supplementation. **(A)** Alpha-diversity for all dogs was assessed using Shannon’s Diversity index, plotted against age, and significance was calculated by univariate linear regression (*p*=0.575). The blue line indicates the regression against the mean (*r*^2^ = 0.010) and shaded gray area indicates the 95% confidence interval. **(B)** Shannon’s Diversity index was calculated for dogs not receiving glucosamine supplements (No) or receiving daily glucosamine (Yes) and significance was calculated using the Kruskal Wallis test (*p*=0.004). Box plots show the median, and first and third quartiles ±1.5 interquartile range (IQR).

To investigate factors influencing gut microbiota diversity in this cohort of sled dogs, we examined the linear mixed effects of age, breed, diet, activity, glucosamine supplementation, injuries, and sex, on bacterial alpha-diversity using Shannon’s diversity index and Faith’s phylogenetic diversity index (Table 1). We found that age (Shannon *p* = 0.012, Faith *p* = 0.071) and dietary glucosamine supplementation (Shannon *p* < 0.001, Faith *p* = 0.005) correlate with variation in alpha-diversity. None of the other variables tested were associated with changes in alpha-diversity metrics. To examine the pattern of alpha-diversity changes across this cohort of dogs, we plotted the Shannon’s diversity value for each dog against age (Figure 1A) and observed that increased age does not correlate with greater microbial diversity by this measure (*p* = 0.575, marginal *r*^2^ = 0.010). We also examined the variation in alpha-diversity by pooling dogs given daily glucosamine supplements and those not receiving glucosamine supplementation. We observed a significant decrease in diversity in the glucosamine-supplemented group (Figure 1B; *p* = 0.004). A re-analysis of the data excluding Sami and Kesha, who upon visual analysis appear to be outliers, retained a significant difference in alpha-diversity associated with glucosamine and age (Supplementary Table S2) indicating that the influences of glucosamine and age on alpha-diversity are not simply due to the oversized effects of these two dogs in the small glucosamine-positive dataset.

**Table 1:** Linear mixed effects analysis reveals differences in alpha-diversity associated with glucosamine supplementation and age.

Variable	Shannon’s Diversity Index	Faith’s Phylogenetic Diversity
Age	0.012^*^	0.071^†^
Breed	0.830	0.226
Diet	0.978	0.750
Pre/post activity	0.473	0.441
Glucosamine	<0.001^*^	0.005^*^
Illness/injury	0.763	0.879
Sex	0.127	0.584

Linear mixed effects were calculated for metadata features including glucosamine supplementation, age, sex, breed, diet, illness/injury, and activity subgroups and were assessed with Shannon’s Diversity Index and Faith’s Phylogenetic Diversity Index alpha-diversity metrics (^*^p < 0.05, ^†^*p* < 0.10). See the Methods section for a definition of each variable.

**Figure 2:**
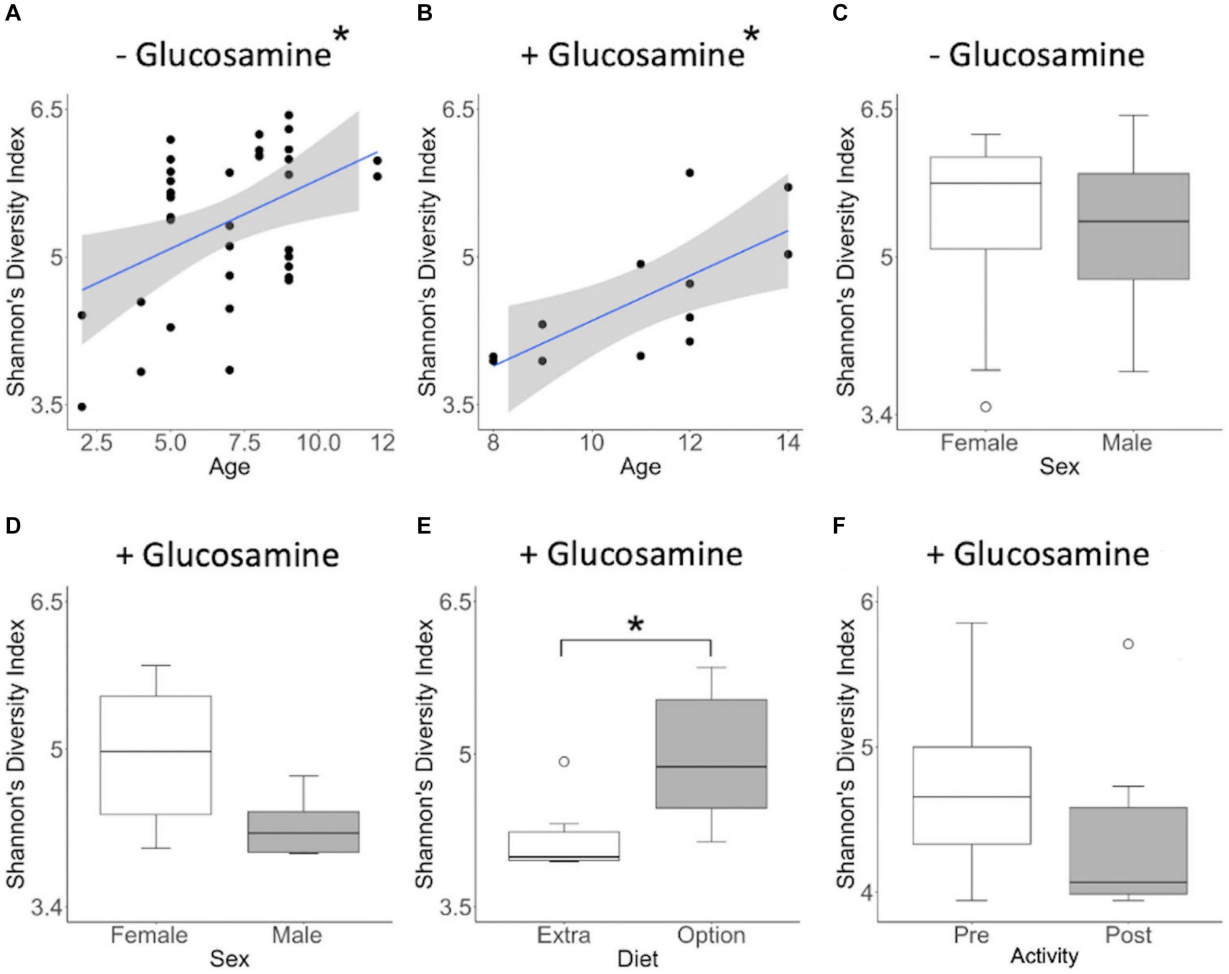
Alpha diversity of gut microbiota varies with age, sex, diet, and activity in the context of glucosamine supplementation. Plots for Shannon’s Diversity index were created for metadata features that were significant in the linear mixed effects analysis for glucosamine subgroups. **(A,B)** Alpha-diversity plotted against age in dogs not receiving glucosamine **(A)** and glucosamine supplemented **(B)**. **(C,D)** Alpha-diversity comparisons by sex in dogs not taking glucosamine **(C)** or receiving glucosamine supplementation **(D)**. **(E)** Alpha-diversity of glucosamine-supplemented dogs on Annamaet Extra and Annamaet Option diets. **(F)** Alpha-diversity pre- and post-activity in dogs receiving glucosamine. Significance was calculated using univariate regression for age and the Kruskal-Wallis test for all other factors (*p < 0.05).

To investigate connections between the tested variables, glucosamine supplementation, and gut microbial diversity, we divided the dataset into glucosamine-supplemented versus no glucosamine subgroups and repeated the linear mixed effects analysis of alpha-diversity metrics (Table 2). We observed significant age-related effects on diversity in dogs not supplemented with glucosamine, as well as a slightly significant effect of sex on Shannon’s diversity. In the smaller group of glucosamine-treated dogs (*n* = 6), we observed significant effects for diet, activity, and sex by at least one measure. To examine these relationships between glucosamine supplementation and microbial diversity more carefully, we plotted alpha-diversity against each variable for cohorts of dogs that were either receiving glucosamine supplements or not glucosamine. We observed a positive relationship between age and alpha diversity in both the absence (*p* = 0.025, *r*^2^ = 0.181; Figure 2A) and presence (*p* = 0.002, *r*^2^ = 0.517; Figure 2B) of glucosamine. In glucosamine-supplemented dogs, we note a trend toward greater alpha-diversity in the gut microbiome in females than males, although this difference was not significant (*p* = 0.055; Figure 2D). In glucosamine-supplemented dogs we observed an increase in diversity in those dogs on a diet of Annamaet salmon option relative to those consuming Annamaet Extra (*p* = 0.025; Figure 2E) but not a significant change in alpha-diversity from pre-activity to post-activity fecal sample collections (*p* = 0.262; Figure 2F).

**Table 2:** Linear mixed effects analyses of dogs lacking or receiving glucosamine supplementation reveal significant differences in alpha-diversity for age, sex, diet, and activity.

No-glucosamine	Shannon’s Diversity	Faith’s Diversity
Age	0.006^*^	0.059^†^
Breed	0.624	0.262
Pre/post-activity	0.763	0.922
Illness/injury	0.330	0.803
Sex	0.047^*^	0.490
Yes-glucosamine		
Age	0.742	0.065^†^
Diet	0.051^†^	0.023^*^
Pre/post-activity	0.368	0.013^*^
Sex	0.002^*^	0.405

Linear mixed effects were calculated for metadata features in glucosamine subgroups and were assessed with Shannon’s Diversity Index and Faith’s Phylogenetic Diversity Index alpha-diversity metrics (^*^p < 0.05, ^†^*p* < 0.10). Diet, breed, and injury variables were eliminated from respective glucosamine analyses due to lack of variance.

**Figure 3:**
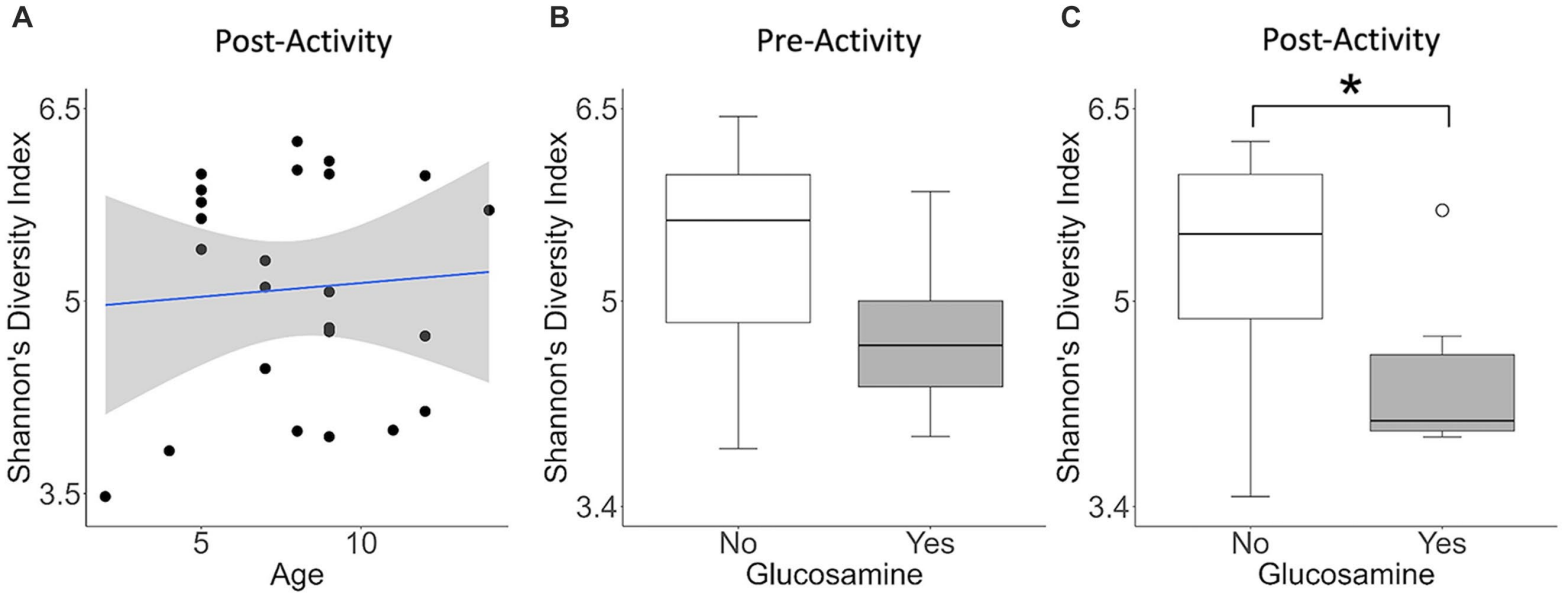
Alpha diversity is lower in glucosamine-supplemented dogs post-activity. Plots for Shannon’s Diversity index were created for metadata features that were significant in the linear mixed effects analysis for activity subgroups (Table 3). Significance was calculated using univariate linear regression for age and the Kruskal-Wallis test for glucosamine supplementation. **(A)** Shannon’s Diversity versus age in dogs post-activity. **(B,C)** Median Shannon’s Diversity comparing dogs receiving glucosamine (Yes) and non-supplemented dogs (No) for pre-activity **(B)** and post-activity **(C)** samples (*p < 0.05).

Because of the differences in alpha-diversity observed before and after activity in glucosamine-supplemented dogs, we further examined the relationship between activity, microbiome composition, and the variables examined, again using linear mixed effects analysis (Table 3). We observed that age correlates with alpha-diversity changes in samples collected post-activity and glucosamine supplementation impacts the gut microbiome in both pre- and post-activity samples. We do not observe an increase in Shannon’s index with increasing age in fecal samples taken from dogs post-activity (*p* = 0.734, *r*^2^ = 0.005; Figure 3A), but do detect lower alpha-diversity in glucosamine-supplemented dogs after activity (*p* = 0.028; Figure 3C).

**Table 3:** Glucosamine supplementation is a significant contributor to alpha-diversity both before and after activity.

Pre-activity	Shannon’s Diversity Index	Faith’s Phylogenetic Diversity
Age	0.188	0.318
Breed	0.977	0.235
Diet	0.744	0.860
Glucosamine	0.032^*^	0.116
Illness/injury	0.976	0.287
Sex	0.497	0.434
Post-activity		
Age	0.037^*^	0.120
Breed	0.607	0.589
Diet	0.758	0.772
Glucosamine	0.011^*^	0.026^*^
Illness/injury	0.565	0.498
Sex	0.153	0.180

Linear mixed effects were calculated for metadata features independently for pre- and post-activity subgroups and were assessed with Shannon’s Diversity Index and Faith’s Phylogenetic Diversity Index alpha-diversity metrics (^*^p < 0.05, ^†^*p* < 0.10).

### Beta-diversity comparison in glucosamine and activity subgroups

**Figure 4:**
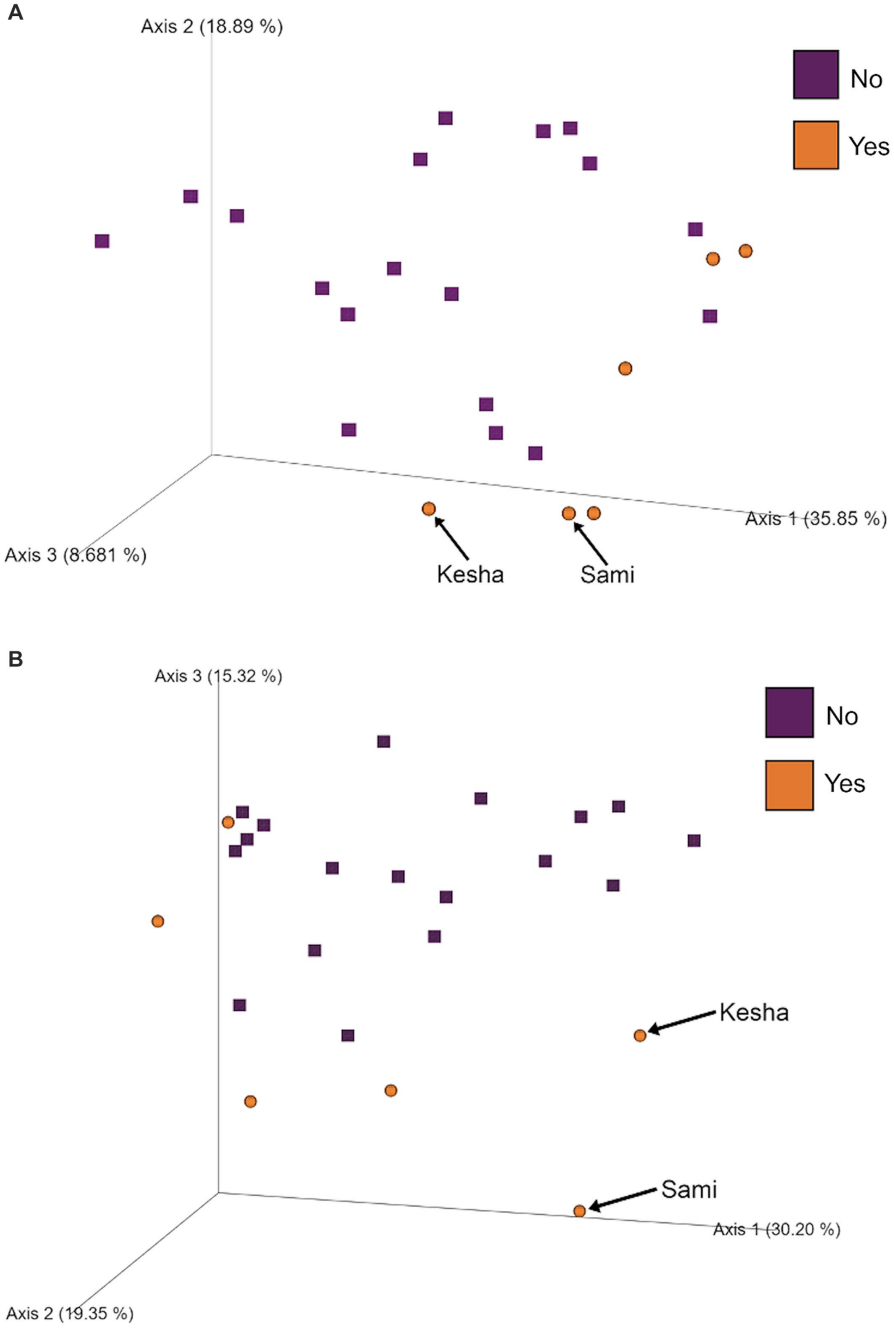
PCoA plots show clustering of dogs based on glucosamine supplementation in pre- and post-activity groups. PCoA plots were created to visualize beta-diversity differences between dogs who did (Yes) and did not (No) receive glucosamine supplementation in **(A)** pre-activity and **(B)** post-activity groups. Arrows indicate dogs Sami and Kesha.

**Figure 5:**
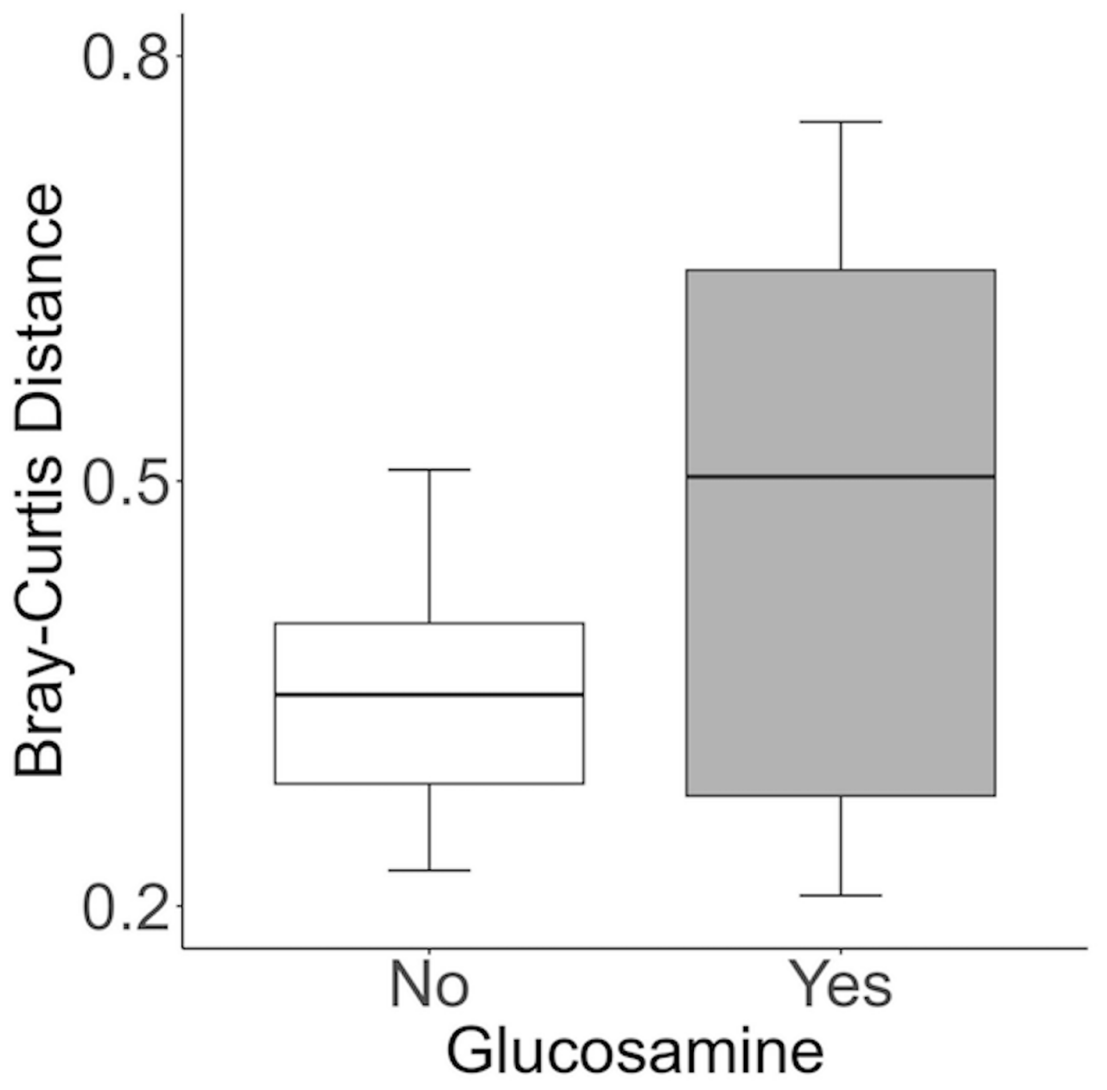
Self-pairwise biodiversity distances between no- and yes-glucosamine groups are not significantly different. Self-pairwise distances were calculated using Bray-Curtis for each dog (*n* = 24) and then a Kruskal Wallis test was performed between distance metrics and glucosamine supplementation status (Yes *n* = 6, No *n* = 18) (*p* = 0.255).

To further investigate which variables correlate with variation in the overall composition of the gut microbiome in this cohort of dogs, we used principal coordinates analysis (PCoA) to visualize differences in Bray-Curtis beta-diversity distance among pre- and post-activity dogs (Figure 4). We observed clustering of the glucosamine-supplemented dogs in both the pre-activity and post-activity analyses. We observed no variation when we compared self-pairwise beta-diversity distances before and after activity between dogs based on glucosamine supplementation status (*p* = 0.255; Figure 5). Because Sami (11 y.o.) and Kesha (12 y.o.) appeared to be potential outliers in both pre- and post-activity PCoAs (Figure 4), we also performed pairwise analyses without the inclusion of these dogs. We again observed no variation in self-pairwise distance between glucosamine treated or untreated dogs when Sami and Kesha were not included in the analysis (*p* = 0.993; Supplementary Figure S1).

**Figure 6:**
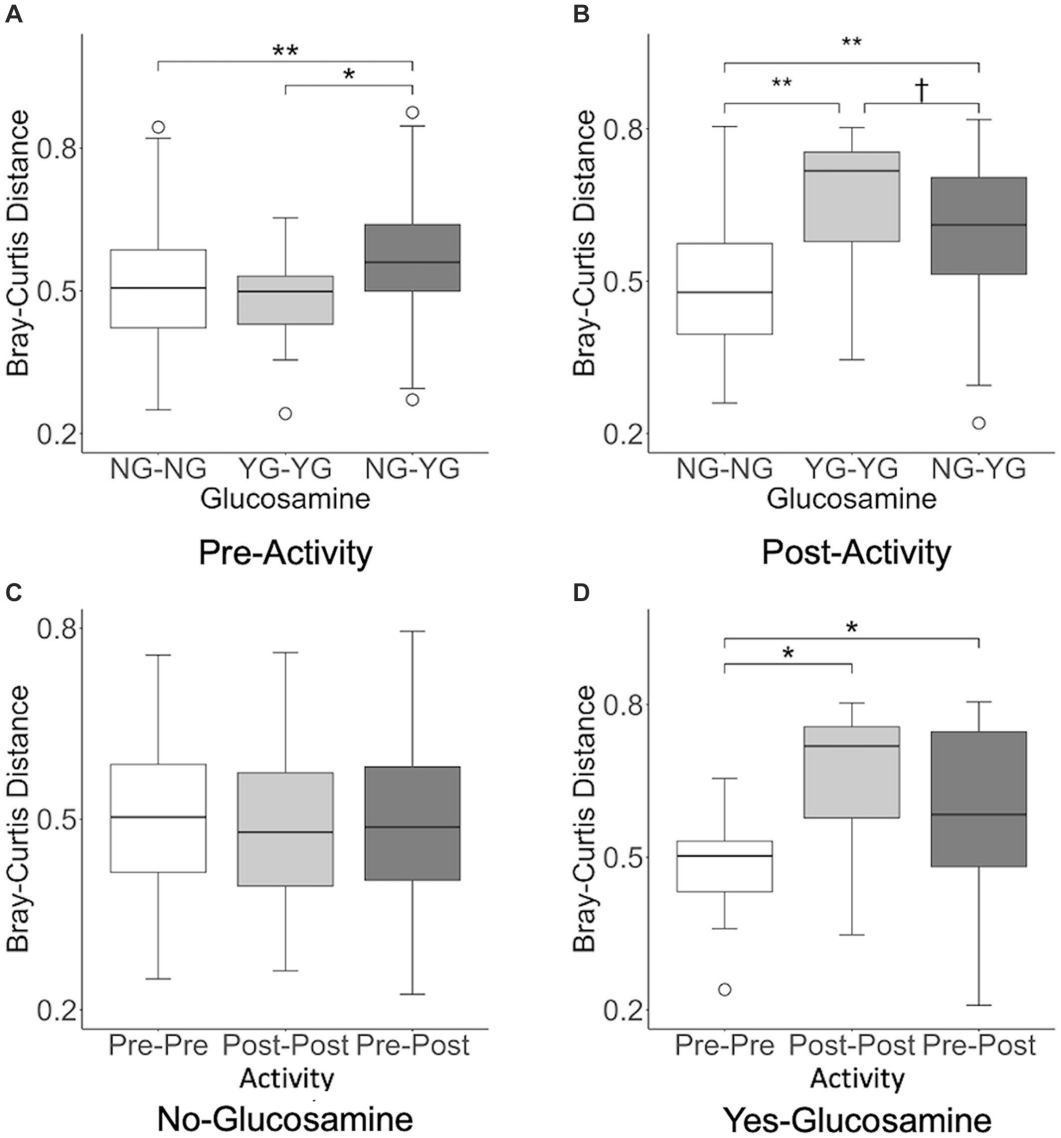
Interindividual pairwise distance analysis reveals variation in beta-diversity distances in pre-activity, post-activity, and glucosamine supplementation groups. Every pairwise Bray-Curtis beta-diversity distance was calculated in **(A)** pre-activity samples, **(B)** post-activity samples, **(C)** no glucosamine samples, and **(D)** glucosamine supplementation samples (^†^*p* < 0.10, *p < 0.05, **p < 0.001 as determined by the Wilcoxon rank-sum test with Benjamini-Hochberg *p*-value correction). NG, no glucosamine; YG, yes glucosamine; Pre, pre-activity; Post, post-activity.

In order to further investigate the correlation between activity, glucosamine supplementation, and variation in the gut microbiome, we performed inter-individual pairwise analyses of beta-diversity distances based on glucosamine supplementation (NG vs. YG) and activity (Pre vs. Post) subgroup data (Figure 6). Analysis of fecal samples collected prior to activity (Figure 6A) indicate significantly greater differences in Bray-Curtis distance between dogs supplemented with glucosamine and those not supplemented (NG-YG) relative to comparisons of non-supplemented dogs to each other (NG-NG; *p* = 0.00082) or supplemented to each other (YG-YG; *p* = 0.019), providing further evidence that glucosamine influences microbiome composition. Post-activity, the NG-YG interindividual distance is again greater than the NG-NG comparisons (Figure 6B; *p* = 8.9E-11). However, the NG-YG distance trends lower than YG-YG distance (*p* = 0.074).

We next examined variation in the gut microbiome pre- and post-activity in the context of glucosamine supplementation. For dogs not taking glucosamine supplementation, pairwise distances between different exercise-related activity subgroups did not differ (Figure 6C). However, for dogs taking a glucosamine supplement, the Pre-Pre distances were shorter than both the Post-Post distances (Figure 6D; *p* = 0.0081) and Pre-Post distances (Figure 6D; *p* = 0.045), again providing evidence for increased microbiome variability with glucosamine supplementation, especially in the context of activity.

We repeated this interindividual pairwise analysis on the dataset excluding Sam and Kesha (Supplementary Figure S2). Before activity, glucosamine supplementation was still associated with greater interindividual pairwise distance between NG-NG and YG-YG (*p* = 0.0018) and between NG-NG and NG-YG (*p* = 0.031). However, in the post-activity group only the NG-NG versus NG-YG differences remained significant (*p* = 0.0015; NG-NG vs. YG-YG: *p* = 0.82). While significant activity-related effects on distance were not observed in glucosamine-treated dogs in the absence of Sami and Kesha, the same general trends in Bray-Curtis distances were retained (Supplementary Figures S2C,D).

### Taxonomic differential abundance analysis

**Figure 7:**
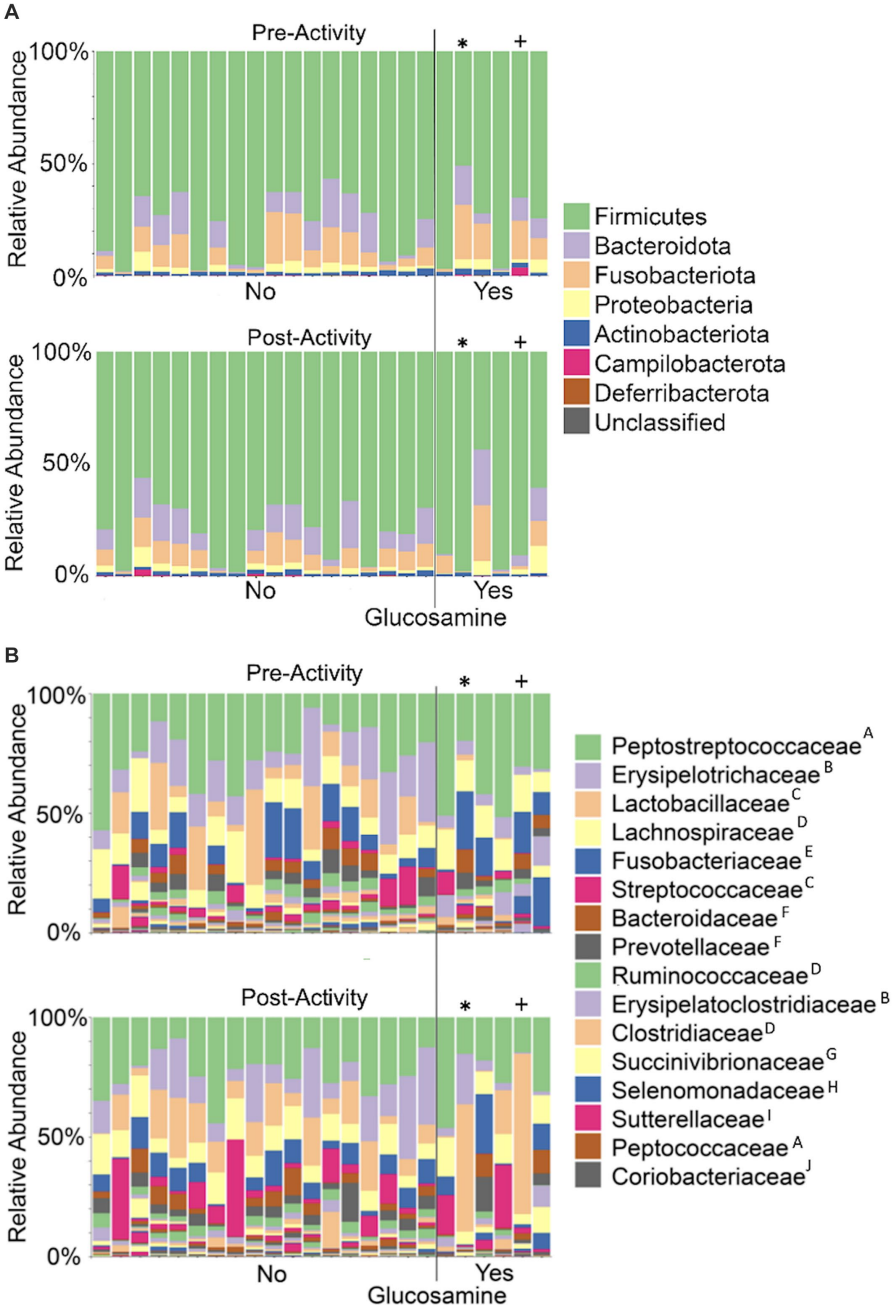
Taxonomic composition of the gut microbiome varies among dogs and within individual dogs pre- and post-activity. Microbial taxa were plotted for relative abundance by phylum **(A)** or family **(B)** for each dog pre-activity (top plot of each panel) or post-activity. Dogs to the left of the vertical line did not receive glucosamine (No) and those to the right received glucosamine supplementation (Yes). (* = Sami; ^+^ = Kesha) Superscripts indicate taxonomic orders: ^A^*Eubacteriales*, ^B^*Erysipelotrichales*, ^C^*Lactobacillales*, ^D^*Clostridiales*, ^E^*Fusobacteriales*, ^F^*Bacteroidales*, ^G^*Aeromonadales*, ^H^*Selenomonadales*, ^I^*Burkholderiales*, ^J^*Coriobacteriales*.

As overall beta-diversity comparisons revealed variation associated with glucosamine supplementation and activity, we sought to determine if specific bacterial taxa were driving these differences. To this end, we first generated taxa bar plots showing relative abundances of the 8 most abundant phyla and found *Firmicutes* to be the most profuse taxa, followed by *Bacteroidota*, and *Fusobacteria* (Figure 7A). Investigating more deeply, we then generated bar plots for each dog’s 16 most abundant families in pre-activity and post-activity samples (Figure 7B). Grouping of these taxa by order reveals that bacteria from the *Eubacteriales, Lactobacillales*, *Clostridiales*, *Bacteriodales*, *Fusobacteriales*, and *Erysipelotrichales* orders were consistently most abundant. There was considerable variability in microbiome composition across all dogs. Visual inspection of changes in relative abundance of families pre- and post-activity suggests an increase in *Streptococcaceae* and a decrease in *Fusobacteriaceae*, although not consistently across all dogs. Two older dogs, Sami and Kesha exhibit a dramatic increase in *Lactobacillaceae* abundance after activity, but this increase is not consistent among most other dogs.

**Figure 8:**
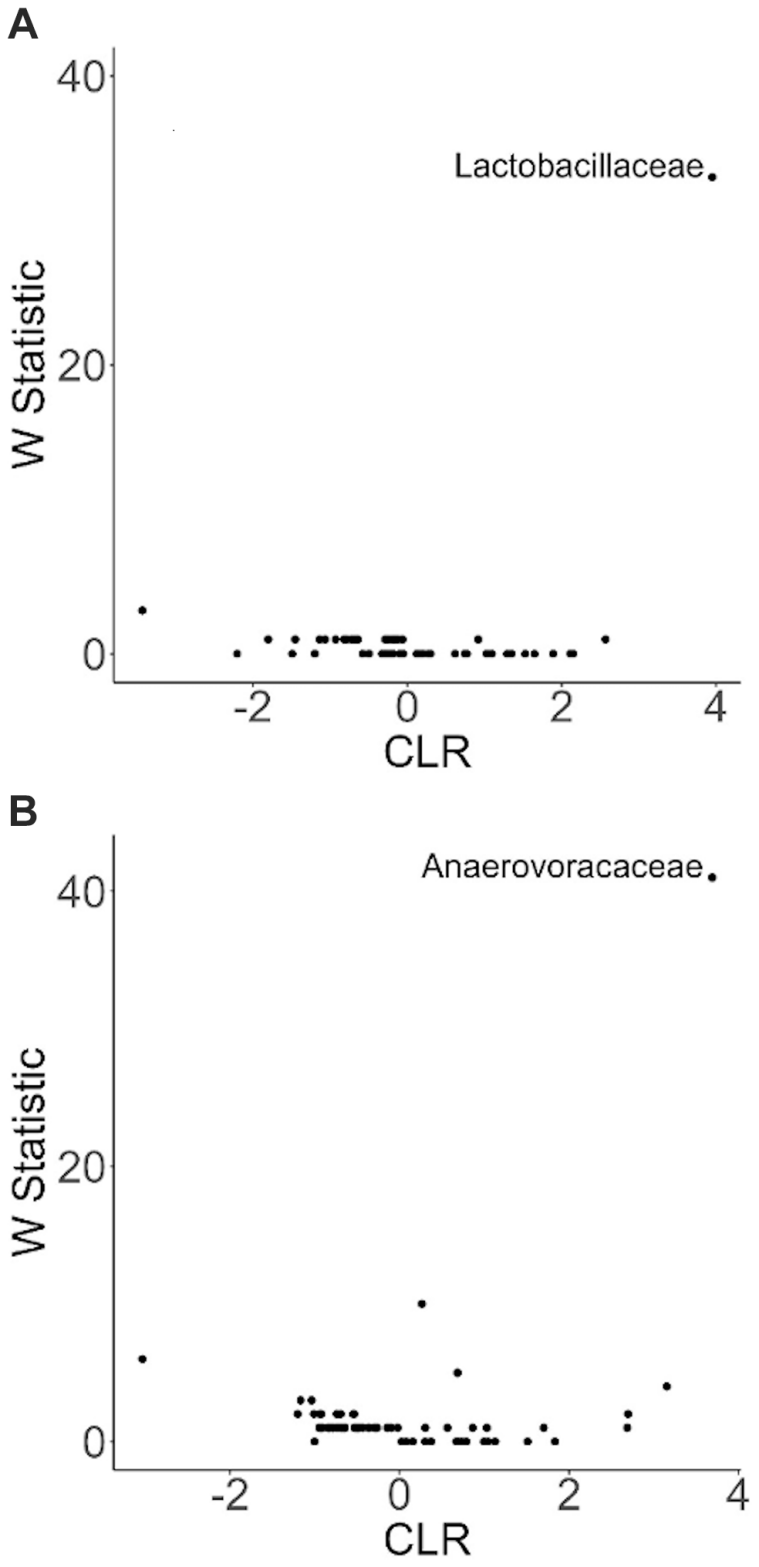
ANCOM plot at the genus level reveals relative abundance variability between glucosamine-treated and untreated dogs for *Lactobacillaceae* and *Anaerovoccaceae* families. ANCOM plots were created and visualized at the family level for the **(A)** pre-activity and **(B)** post-activity subgroups, using glucosamine as the comparison variable.

**Figure 9:**
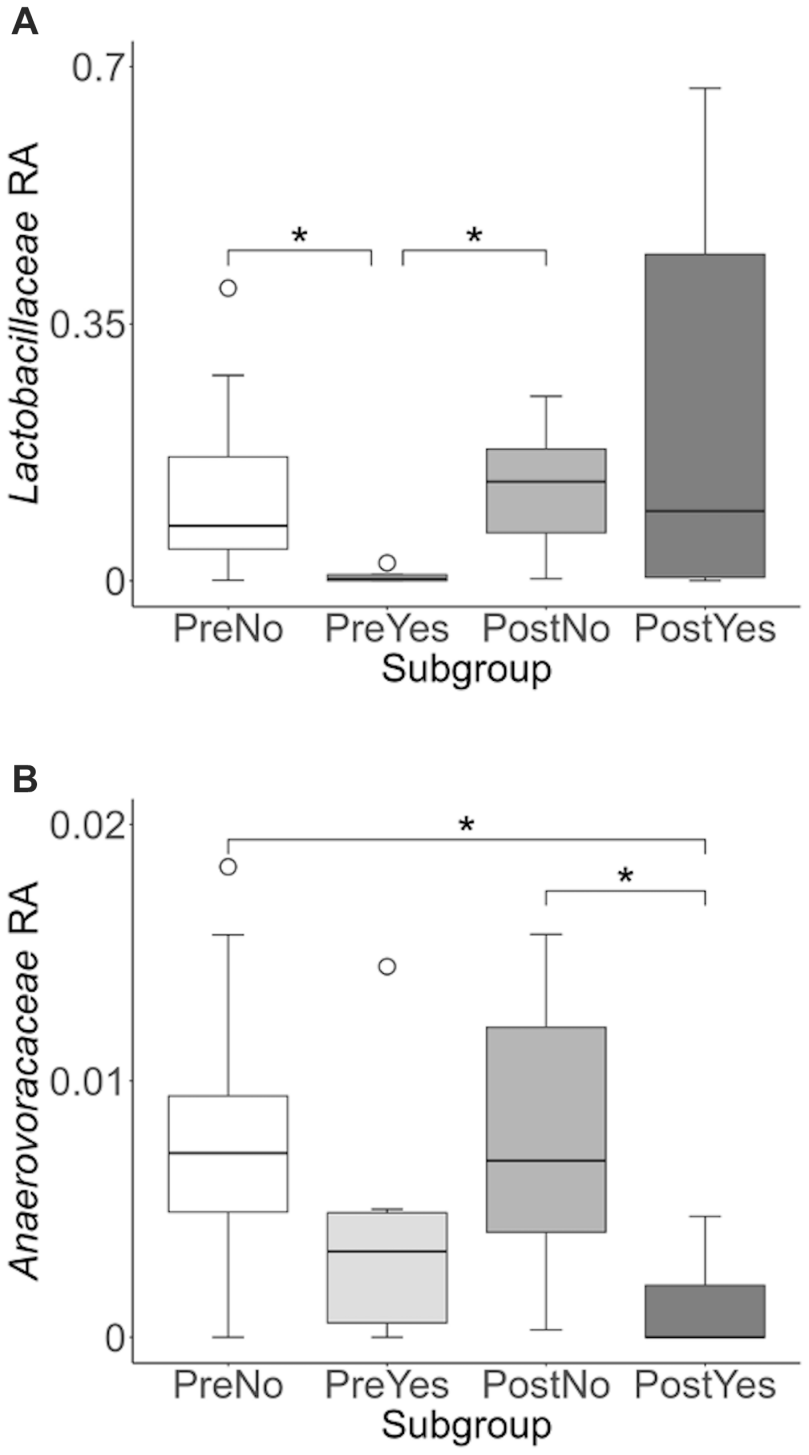
*Lactobacillaceae* and *Anaerovoracaceae* taxa abundance plots reveals differences in relative abundance by population subgroups. Relative abundance analysis was performed in **(A)** *Lactobacillaceae* and **(B)** *Anaerovoracaceae* families for pre-activity no-glucosamine (PreNo), pre-activity yes-glucosamine (PreYes), post-activity no-glucosamine (PostNo), and post-activity and yes-glucosamine (PostYes) population subgroups. (*p < 0.05 as determined by the Wilcoxon rank-sum test with Benjamini-Hochberg *p*-value correction).

To more closely examine which bacterial taxa exhibit variability among sled dogs, we performed an Analysis of Composition of Microbiomes (ANCOM) (65). ANCOM revealed that *Lactobacillaceae* and *Anaerovoccaceae* families exhibited the greatest variation in relative abundance among sled dogs (Figure 8). To more carefully examine the relationship between activity, glucosamine supplementation, and the variation in *Lactobacillaceae* and *Anaerovoracaceae*, we observed the relative change in abundance of each taxa under conditions of pre- and post-activity in dogs grouped by glucosamine supplementation. *Lactobacillaceae* was decreased in glucosamine-supplemented dogs before activity relative to the abundance levels in dogs not receiving glucosamine before and after activity (Figure 9A). Much greater *Lactobacillaceae* variability was observed in glucosamine-supplemented dogs post-activity, although the observed differences from dogs not taking glucosamine were not statistically significant. Given the increased relative abundance of *Lactobacillaceae* in Sami and Kesha after activity (see Figure 7), we repeated the analysis of relative abundance on the cohort minus these dogs (Supplementary Figure S3A). In the absence of data from Sami and Kesha, we still observe significantly lower *Lactobacillaceae* in glucosamine-supplemented dogs pre-activity relative to dogs not receiving glucosamine, and also see a trend toward lower *Lactobacillaceae* post-activity in glucosamine-treated dogs. However, this number is not statistically significant.

*Anaerovoracaceae* abundance was decreased in glucosamine-supplemented dogs after activity, compared to dogs not receiving glucosamine, both before and after activity (Figure 9B). The same pattern of significant differences in *Anaerovoracaceae* were retained even when Sami and Kesha were excluded from analysis (Supplementary Figure S3B).

**Figure 10:**
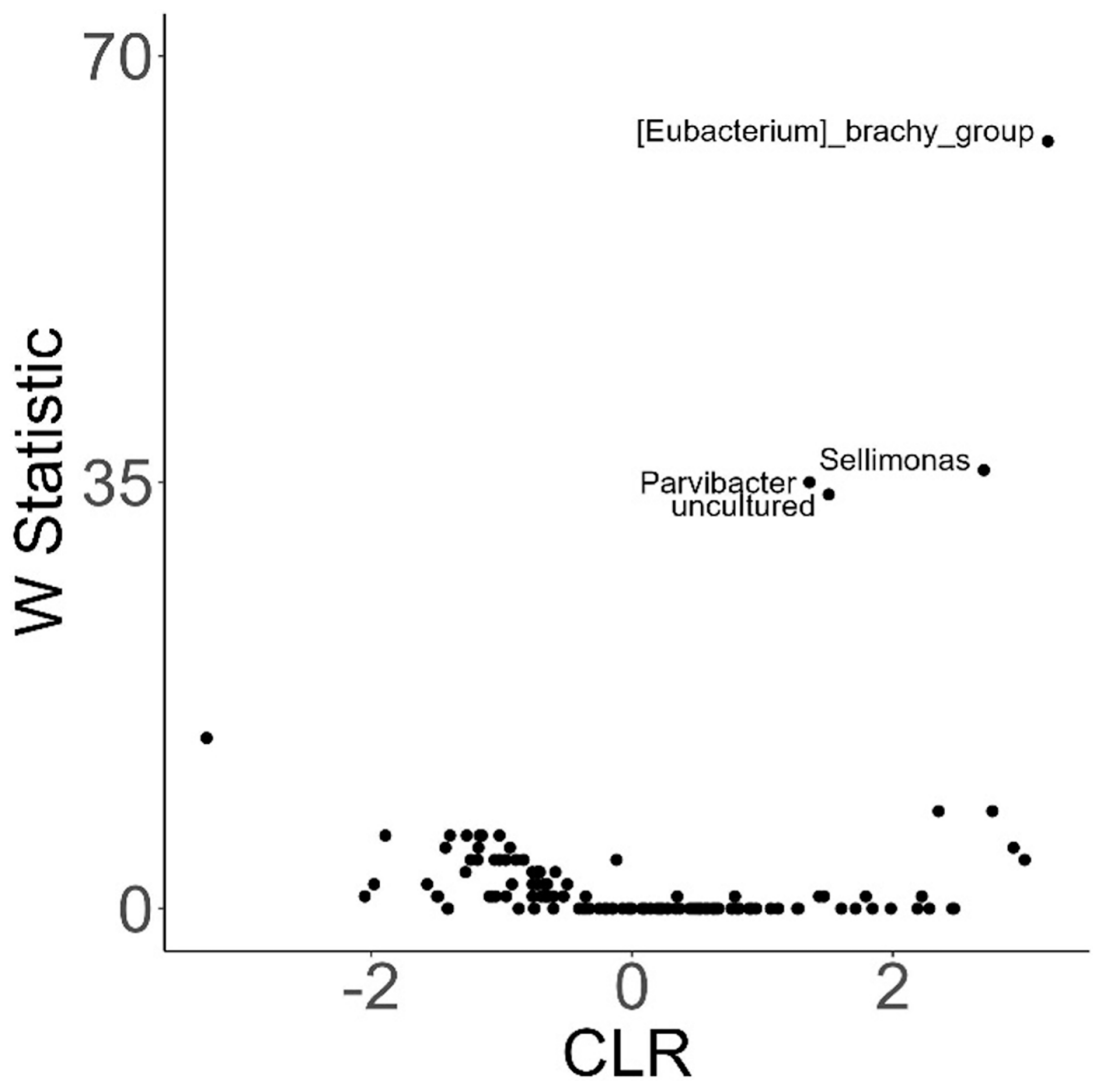
ANCOM plot at the genus level reveals relative abundance variability between glucosamine-treated and untreated dogs for *Eubacterium [brachy]*, *Sellimonas*, *Parvibacter*, and an uncultured genus belonging to the same *Eggerthellaceae* family. ANCOM plots were created and visualized at the genus level for dogs post-activity, using glucosamine supplementation as the comparison variable.

**Figure 11:**
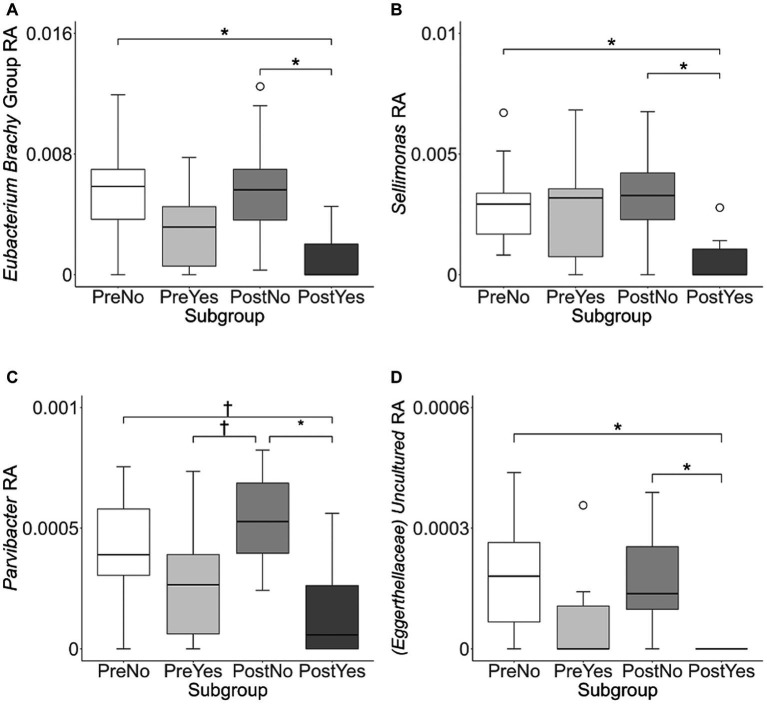
*Eubacterium* [*brachy*], *Sellimonus*, *Parvibacter*, and uncultured genera taxa abundance plots reveals differences in relative abundance by population subgroups. Relative abundance analysis was performed in **(A)** *Eubacterium* [*brachy*] **(B)** *Sellimonas* **(C)** *Parvibacter*, and **(D)** Uncultured genera from the same family as *Parvibacter* (*Eggerthellaceae*) for exercise and glucosamine supplementation subgroups. *Eubacterium* [*brachy*] includes the genus (*Eubacterium*), and the analytical tool also gave the species (*brachy*) when this analysis was performed at the genus level. (PreNo = pre-activity no-glucosamine; PreYes = pre-activity yes-glucosamine; PostNo = post-activity no-glucosamine; PostYes = post-activity and yes-glucosamine subgroups. *p < 0.05 and †*p* < 0.10 as determined by the Wilcoxon rank-sum test with Benjamini-Hochberg *p*-value correction).

To investigate variation in the microbiome at a finer taxonomic level, ANCOM analysis was repeated using genus as the taxonomic criteria. At the genus level, our ANCOM plot revealed that *Eubacterium* [*brachy*], *Sellimonas*, *Parvibacter*, and an uncultured genus belonging to the same *Eggerthellaceae* family as *Parvibacter* clearly exhibited the greatest abundance variability among dogs, relative to other taxa (Figure 10). We again investigated the importance of activity and glucosamine supplementation on the varying abundances of these genera (Figure 11). *Eubacterium* [*brachy*] and *Sellimonas* abundances were lower in glucosamine-supplemented dogs post-activity relative to the abundances of pre- and post-activity dogs that did not receive glucosamine (Figures 11A,B). *Parvibacter* abundance was significantly lower after activity in glucosamine-supplemented dogs, compared to after activity in dogs that did not receive glucosamine and trended toward lower before activity, as well (Figure 11C). The uncultured genus belonging to the same *Eggerthellaceae* family as *Parvibacter* exhibited lower abundance in glucosamine-supplemented dogs after activity, compared to abundance in dogs that did not receive glucosamine, in both pre- and post-activity groups (Figure 11D). In genus-level analysis without Sami and Kesha the same trends were observed for *Eubacterium* [*brachy*], *Sellimonus*, and the uncultured genus, but not for *Parvibacter* (Supplementary Figure S4).

## Discussion

In this study, we sought to identify factors that correlate with variation in the fecal microbiome of a cohort of active sled dogs. We observe that younger age and dietary glucosamine supplementation are associated with reduced alpha-diversity. Variations in sex and activity also correlate with differences in alpha-diversity, specifically among those dogs receiving glucosamine supplementation. Beta-diversity analysis using PCoA reveals clustering among glucosamine supplemented dogs and inter-individual pairwise distances are longest between dogs of different glucosamine ingestion statuses. Taxonomic analysis at the family level reveals that sled dogs taking glucosamine have decreased *Lactobacillaceae* and *Anaerovoracaceae*. At the genus level, *Eubacterium brachy*, *Sellimonas*, *Parvibacter*, and an unclassified genus belonging to the same family as *Parvibacter* exhibited reduced abundances in glucosamine-supplemented dogs, particularly after activity. Our data provide evidence that glucosamine supplementation and exercise-related activity alter the gut microbiome in active sled dogs.

### Alpha-diversity

The majority of significant variables in our alpha-diversity analysis were identified exclusively by Shannon’s Diversity metric. This metric considers both species richness and relative abundance, while Faith’s phylogenetic diversity, our other metric, reflects the number of phylogenetic units in a sample. Therefore, these findings suggest that most of these factors contribute to differences in relative abundances amid a relatively stable environment of microbial species richness. We observed variations in alpha-diversity for dogs that received glucosamine supplementation in the overall dataset and in the pre- and post-activity subgroups. Our results agree with previous observations that dogs supplemented with glucosamine or similar sulfated carbohydrates exhibit a significant decrease in alpha-diversity (44, 45, 67). Here, we provide evidence that other lifestyle factors, including activity and age, contribute to these changes.

Activity was associated with reduced alpha-diversity in the subgroup of dogs receiving glucosamine, according to Faith’s phylogenetic diversity. Previously, Tysnes et al. (24) found that alpha-diversity in non-supplemented sled dogs did not differ pre- and post-race. Similar to our findings above, it was also found that age had an important role in changes in gut composition after exercise (24). Interestingly, the removal of our two oldest dogs from the glucosamine subgroup eliminated the significant effect of activity. For this reason, it is unclear how much of this association is due to glucosamine and how much is due to age-related effects. Moreover, human studies have yielded conflicting findings but suggest that the effects of exercise are also highly dependent on the duration and intensity of exercise (68). Since activity was not uniform among dogs, this may also have a confounding role in our findings, although it was previously shown that training duration did not affect alpha-diversity in hunting dogs (69).

Our mixed effects analyses indicate that sled dogs in our cohort have a significant variation in alpha-diversity correlating with age, both overall and within the post-activity dogs and dogs not taking glucosamine. Age was not associated with alpha-diversity in the yes-glucosamine subgroup, possibly suggesting that glucosamine affects microbial richness in ways that obscure age-related effects. Plotting alpha-diversity with age revealed a trend toward an increase in Shannon’s Diversity index as dogs age, and this trend was retained in dogs taking glucosamine. Interestingly, previous studies in dogs have observed a decrease in gut microbiome alpha-diversity with age (70, 71) or no overall trend in diversity (72). However, these inconsistencies could be explained by the sample population, as the age range and health condition of sampled dogs strongly influence overall findings. Multiple studies find correlation between a decrease in alpha-diversity and advancing age in humans (73–76) see Martino et al. (77). However, more recent analyses suggest that the relationship between aging and microbiome complexity may be more complicated. Sato et al. (78) observe that centenarians actually have increased alpha-diversity compared to a young control population as calculated by Shannon index. Studies taking into account health status across a broad age range suggest that there is little difference in microbiome complexity between healthy young and old groups (79), or observe stability or increases in Shannon diversity in very old participants (80, 81). Health status and environmental factors are likely better predictors of microbiome composition than age (82, 83), or at least must be taken into account when considering the complex interactions between aging and gut microbiota (84, 85). The dogs in our study may retain high alpha-diversity at an older age as a result of some lifestyle factor such as exercise, communal living, diet, or health intervention.

### Beta-diversity

PCoA analysis examining beta-diversity revealed clustering of the glucosamine supplemented cohort of dogs. This was reinforced by our inter-individual pairwise distance analysis, as Bray-Curtis distances are greatest when comparing glucosamine-treated dogs with untreated prior to activity. These findings suggest that glucosamine had clear effects on beta-diversity. These findings are supported in humans by Navarro et al. (67) who supplemented individuals with both glucosamine and chondroitin, a polymeric sulfated glycosaminoglycan. In a small study on humans, Moon et al. (45) supplemented individuals with glucosamine for 3 weeks at 3,000 mg and did not find significant differences in beta-diversity in supplemented individuals, despite changes in individual taxa. Interestingly, we found a greater Bray-Curtis distance after activity between dogs who received glucosamine compared to those without supplementation, which is contrary to the notion that glucosamine fosters a specific gut microbial environment conducive to anti-inflammatory symptoms. However, this difference largely disappeared upon removal of two older, glucosamine-supplemented dogs (Sami and Kesha) from our pairwise analysis. While our data reveal significant differences in both alpha and beta-diversity correlating with glucosamine supplementation, further studies will help clarify the mechanism of these alterations and their connections to activity and other life history factors.

### Differential abundance of specific taxa

As part of our analysis, we examined changes in abundance across all taxa of bacteria and archaea detected through sequencing of the V4 variable region of 16S rRNA from glucosamine-supplemented and non-supplemented dogs. Glucosamine, a sulfated monosaccharide, can thus serve as a substrate for sulfate-reducing bacteria (43). One model for potential beneficial effects of glucosamine treatment is that the H_2_S produced by sulfate-reducing bacteria may have anti-inflammatory and cytoprotective effects (86–88), and may be involved in the resolution of tissue injury in the gut (88, 89). However, our data analysis did not reveal increased abundance of sulfate-reducing bacteria among taxa in glucosamine-treated dogs.

While we did not observe increases in sulfate-reducing taxa, we did observe the differential abundance of taxa implicated in inflammatory and anti-inflammatory processes at the family and genus levels. At the family level, we observed the reduction of *Lactobacillaceae* in glucosamine-treated dogs prior to activity and a significant variation in abundance post- activity. *Lactobacillaceae rhamnosus* has been shown to exert important anti-inflammatory effects in the gut (90), and is used as a therapeutic probiotic strain (91). *Lactobacillus* abundance has been associated with both inflammatory bowel diseases and chronic rheumatic diseases (92), and *Lactobacillaceae* has emerged as a potential biomarker for systemic inflammation (93). Indeed, recent research in Alaskan sled dogs found that dogs exhibited intestinal erosions following exercise. However, there was no difference in pro-inflammatory cytokines in these sled dogs (22). Therefore, the increase in pro-inflammatory taxa that we observe after activity in supplemented dogs may represent a more efficient response to inflammation, not exerting its effect by preventing inflammation in the first place.

We also observed differences in *Anaerovoccaceae* between glucosamine-treated and untreated dogs. While *Anaerovoccaceae* abundance did not significantly differ with or without glucosamine in the pre-activity group, it trended lower. And the abundance of this taxa was significantly reduced post-activity in glucosamine-supplemented dogs. Previously, physical activity has been positively associated with *Anaerovoccaceae* and negatively associated with propionate abundance (94). Propionate is a short-chain fatty acid (SCFA) known to have anti-inflammatory effects (95). Therefore, it seems possible that *Anaerovoccaceae* is a pro-inflammatory bacterial family, involved in suppressing inflammatory response.

At the genus level, we also observed the reduction of *Parvibacter*, *Eubacterium* [*brachy*], and *Sellimonus* after activity in glucosomine-treated dogs. *Parvibacter* abundance is also inversely associated with propionate abundance (96), so it may also be suppressed by *Lactobacillaceae* and/or sulfate-reducing bacteria. *Eubacterium brachy* was initially isolated from subgingival samples of patients with moderate and severe periodontitis (97), it has recently been found to have a potential role in metabolic syndrome in the microflora (98). *Eubacterium brachy*, among other species belonging to the *Eubacteria* genus, may secrete virulence factors that are associated with the degradation of host tissue as it localizes to infected sites (99). *Eubacterium brachy* could also be suppressed by *Lactobacillaceae* or sulfate-reducing bacteria following activity, which would mediate decreases in inflammatory response.

*Sellimonus* abundance has previously been associated with several inflammatory disorders including rheumatoid arthritis, ankylosing spondylitis, liver cirrhosis, and systemic-onset juvenile arthritis (100–102). Recent examination of *Sellimonas* has revealed that this genus may be a biomarker for gut homeostasis in diseased patients (103). *S. intestinalis* was sequenced and found to include genes involved in SCFA production (103), which is involved in gut barrier integrity, metabolism, immune system regulation, and regulation of inflammatory response (104). *Sellimonus* belongs to the *Lachnospiraceae* family, which contains many taxa involved in SCFA production and taxa that have been implicated in intra- and extra-intestinal diseases (105). Previously, it has been shown that acids produced by *Lactobacillaceae* species inhibit the growth of *Lachnospiraceae* taxa (106). While the mechanism of this interaction has not yet been delineated (106), this regulatory effect may demonstrate a decreased need for this reactionary anti-inflammatory response since there are more *Lactobacillaceae* taxa present to mitigate the more immediate inflammatory effects of exercise.

### Limitations

Our findings must be considered in light of the following limitations. First, this study is a retrospective study utilizing a small dataset of 24 dogs, which may limit the generalizability of our findings. Those dogs receiving glucosamine supplementation ingested it in different forms (glucosamine and cosequin tablets and chews, some of which may contain ascorbic acid and/or methylsufonylmethane) and in different quantities. In addition, dogs with a wide variation in age were included in the study and those on glucosamine supplementation skewed older than those not taking glucosamine. Activity was also not equivalent for all dogs, as some pulled tour groups over a three-day span while others ran independently of sleds. However, all dogs engaged in physical activity over this span, potentially providing the effects that accompany endurance activity (107). With a larger sample size in the future, we might better be able to analyze the influence of sex and age on microbiome composition, and also include whether dogs are intact, as spayed or neutered dogs may have variations in hormone levels that could influence microbial composition (50, 108). Additionally, inter-individual genetic variation between dogs that could have contributed to variation in gut microbial composition and influenced our results. While insights suggest that Inuit dogs have retained genetic markers unique to their indigenous history, these breeds have occupied overlapping geographical regions as sled dogs since the European colonization of the Arctic (109, 110). Multiple studies have shown that gut microbial composition depends on environment (18, 111), which may contribute to similarities in microbiota among dogs in this study. Careful longitudinal analysis of multiple samples from before and after intense activity and with and without glucosamine supplementation will likely provide further insights into the influence of these variables on microbiome composition.

## Conclusion

Our findings suggest that glucosamine supplementation impacts gut microbiome composition in this population of sled dogs, especially in the context of periods of greater activity. Sex, diet, and age also correlate with changes in gut microbiota, with an increasing complexity of microbes with increasing age. These results suggest that further study is important for understanding fully the effects of glucosamine supplementation in active dogs.

## Data availability statement

The datasets presented in this study can be found in online repositories. The names of the repository/repositories and accession number(s) are available at the following link: http://www.ncbi.nlm.nih.gov/bioproject/1060330.

## Ethics statement

The animal study was approved by Colgate University Institutional Animal Care and Use Committee. The study was conducted in accordance with the local legislation and institutional requirements.

## Author contributions

DW: Data curation, Formal analysis, Investigation, Methodology, Visualization, Writing – review & editing. WR: Investigation, Visualization, Writing – original draft. KM: Data curation, Formal analysis, Investigation, Methodology, Visualization, Writing – review & editing. VD: Conceptualization, Investigation, Writing – review & editing. AA: Conceptualization, Formal analysis, Methodology, Project administration, Resources, Supervision, Visualization, Writing – review & editing. KDB: Conceptualization, Funding acquisition, Investigation, Project administration, Resources, Supervision, Writing – review & editing.

## Footnotes

^1^https://www.qiagen.com/us/products/discovery-and-translational-research/dna-rna-purification/dna-purification/microbial-dna/dneasy-powersoil-pro-kit

^2^earthmicrobiome.org/protocols-and-standards/16s
