# Supplementary material for: Analysis of the gut microbiome in sled dogs reveals glucosamine- and activity-related effects on gut microbial composition
Source: Front Vet Sci. 2024 Feb 7;11:1272711. doi: 10.3389/fvets.2024.1272711 (PMC10879321; doi:10.3389/fvets.2024.1272711)
Supplement: Supplementary file 1 [file Data_Sheet_1.docx]

**Supplementary Table 1.** Information regarding each sample included in analysis. ‘Date collected’ is the date of original fecal sample collection. ‘No. reads’ indicates the number of 16S rDNA sequence reads included in the FastQ file for each sample. ‘16S PCR conc’ indicates the concentration of DNA in each sample after the initial 16S PCR amplification in ng/ul. This sample was then diluted and used for sequencing as described in the Materials and Methods.

| Sample name | Dog | Date collected | Pre/post activity | No. Reads | 16S PCR conc (ng/ul) |
| --- | --- | --- | --- | --- | --- |
| MN10Mar_1 | Koyuk | 1/27/22 | Pre | 189111 | 7.26 |
| MN10Mar_2 | Toklat | 1/27/22 | Pre | 158342 | 10.8 |
| MN10Mar_4 | Mooz | 1/27/22 | Pre | 191006 | 8.52 |
| MN10Mar_5 | Akiak | 1/27/22 | Pre | 169439 | 10.1 |
| MN10Mar_6 | Urho | 1/27/22 | Pre | 160024 | 12.3 |
| MN10Mar_6dupl | Urho | 1/27/22 | Pre | 149396 | 5.33 |
| MN10Mar_7 | Newton | 1/27/22 | Pre | 182886 | 11.3 |
| MN10Mar_8 | Kylla | 1/27/22 | Pre | 131018 | 11.4 |
| MN10Mar_9 | Finn | 1/27/22 | Pre | 163388 | 12 |
| MN1Mar_1 | blank | n/a | n/a | 83652 | 2.05 |
| MN1Mar_10 | Kesha | 1/31/22 | Post | 137634 | 16.1 |
| MN1Mar_11 | Toklat | 1/31/22 | Post | 167748 | 15.8 |
| MN1Mar_12 | Tulugak | 1/31/22 | Post | 135833 | 8.77 |
| MN1Mar_13 | Amaroq | 1/31/22 | Post | 188309 | 14.6 |
| MN1Mar_14 | Susi | 1/31/22 | Post | 167844 | 16.1 |
| MN1Mar_15 | Lena | 1/31/22 | Post | 201803 | 10.7 |
| MN1Mar_16 | Lemmikki | 1/31/22 | Post | 173012 | 18.1 |
| MN1Mar_2 | Ukho | 1/31/22 | Post | 196939 | 13.2 |
| MN1Mar_2dupl | Ukho | 1/31/22 | Post | 147581 | 5.5 |
| MN1Mar_3 | Mooz | 1/31/22 | Post | 179968 | 10.8 |
| MN1Mar_4 | Sitka | 1/31/22 | Post | 186358 | 16.9 |
| MN1Mar_5 | Finn | 1/31/22 | Post | 188852 | 15.9 |
| MN1Mar_6 | Suen | 1/31/22 | Post | 179964 | 9.33 |
| MN1Mar_7 | Newton | 1/31/22 | Post | 160158 | 15.3 |
| MN1Mar_7dupl | Newton | 1/31/22 | Post | 186251 | 9.67 |
| MN1Mar_8 | Sigurd | 1/31/22 | Post | 162201 | 7.83 |
| MN1Mar_9 | Kylla | 1/31/22 | Post | 163241 | 13.7 |
| MN28Feb_2 | Mukwah | 1/31/22 | Post | 179301 | 16.6 |
| MN28Feb_3 | Nikolai | 1/31/22 | Post | 173362 | 4.24 |
| MN28Feb_4 | Foxy | 1/31/22 | Post | 200828 | 14.5 |
| MN28Feb_5 | Willow | 1/31/22 | Post | 195492 | 14.2 |
| MN28Feb_6 | Akiak | 1/31/22 | Post | 181103 | 15.9 |
| MN28Feb_7 | Wing | 1/31/22 | Post | 157235 | 7.01 |
| MN28Feb_8 | Nome | 1/31/22 | Post | 189106 | 13.7 |
| MN3Mar_1 | E. coli pos | n/a | n/a | 175875 | 21.5 |
| MN3Mar_2 | Sami | 1/31/22 | Post | 189271 | 19.4 |
| MN3Mar_3 | Monk | 1/31/22 | Post | 166439 | 15 |
| MN3Mar_4 | Akiak | 1/31/22 | Post | 165114 | 17.6 |
| MN3Mar_5 | Tahti | 1/31/22 | Post | 160382 | 18.2 |
| MN3Mar_6 | Olga | 1/31/22 | Post | 223300 | 16.9 |
| MN3Mar_6dupl | Olga | 1/31/22 | Post | 202687 | 12.1 |
| MN3Mar_7 | Osa | 1/31/22 | Post | 206992 | 16.4 |
| MN3Mar_8 | Susi | 1/27/22 | Pre | 207182 | 13.7 |
| MN8Mar_1 | Monk | 1/27/22 | Pre | 175251 | 11.7 |
| MN8Mar_1dupl | Monk | 1/27/22 | Pre | 162616 | 6.25 |
| MN8Mar_2 | Foxy | 1/27/22 | Pre | 205559 | 8.32 |
| MN8Mar_3 | Tulugak | 1/27/22 | Pre | 180184 | 10.1 |
| MN8Mar_4 | Lemmikki | 1/27/22 | Pre | 193020 | 11.5 |
| MN8Mar_4dupl | Lemmikki | 1/27/22 | Pre | 198613 | 10.2 |
| MN8Mar_5 | Wing | 1/27/22 | Pre | 183724 | 7.08 |
| MN8Mar_6 | Mukwah | 1/27/22 | Pre | 161694 | 8.29 |
| MN8Mar_7 | Kesha | 1/27/22 | Pre | 159784 | 10.3 |
| MN8Mar_8 | Willow | 1/27/22 | Pre | 145633 | 11.8 |
| MN9Mar_1 | Amaroq | 1/27/22 | Pre | 163988 | 8.89 |
| MN9Mar_2 | Osa | 1/27/22 | Pre | 145363 | 11.8 |
| MN9Mar_3 | Lena | 1/27/22 | Pre | 169152 | 10.4 |
| MN9Mar_4 | Suen | 1/27/22 | Pre | 175155 | 10.7 |
| MN9Mar_5 | Sami | 1/27/22 | Pre | 189440 | 5.97 |
| MN9Mar_5dupl | Sami | 1/27/22 | Pre | 137602 | 6.26 |
| MN9Mar_6 | Sigurd | 1/27/22 | Pre | 184018 | 8.86 |
| MN9Mar_7 | Nikolai | 1/27/22 | Pre | 162061 | 11.1 |
| MN9Mar_8 | Sitka | 1/27/22 | Pre | 210996 | 8.27 |
| MNnofeces | blank | n/a | n/a | 28313 | 0.99 |

**Supplementary Table 2.** Linear mixed effects analysis reveals differences in alpha-diversity associated with glucosamine and age in a dataset lacking dogs Sami and Kesha. With Sami and Kesha removed, linear mixed effects were calculated for metadata features including glucosamine, age, sex, breed, diet, illness/injury, and activity subgroups and were assessed with Shannon’s Diversity Index and Faith’s Phylogenetic Index alpha-diversity metrics. ( ***** = p<0.05, † = p<0.10).

| Variable | Shannon’s Diversity Index | Faith’s Phylogenetic Diversity |
| --- | --- | --- |
| Age | 0.004* | 0.041* |
| Breed | 0.612 | 0.239 |
| Diet | 0.731 | 0.235 |
| Activity | 0.909 | 0.626 |
| Glucosamine | 0.022* | 0.066^†^ |
| Illness/Injury | 0.471 | 0.876 |
| Sex | 0.041 | 0.423 |
|  |  |  |


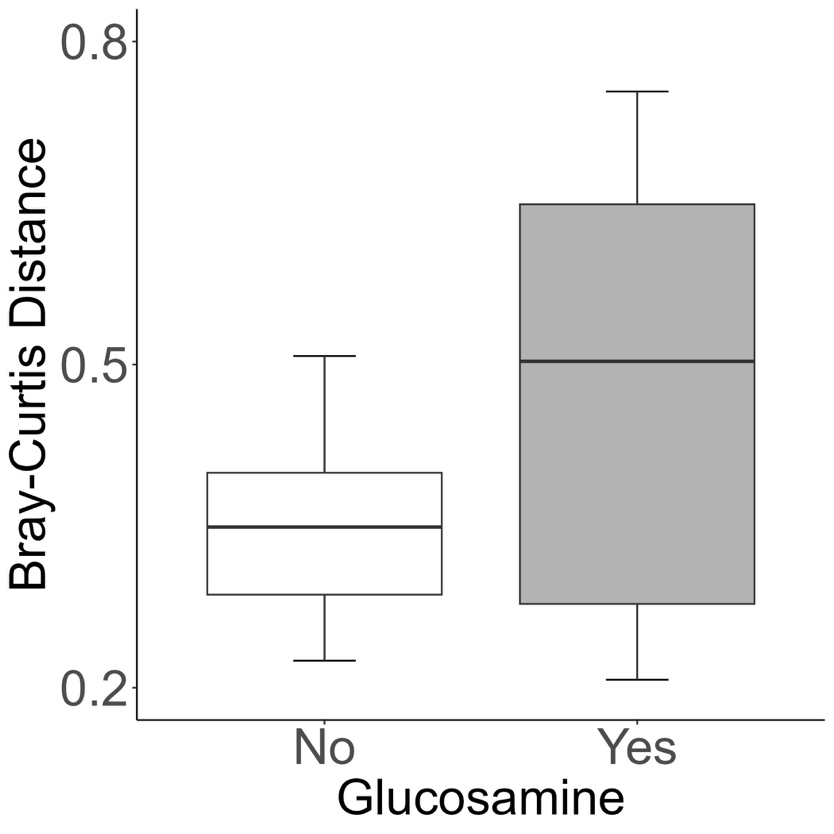


**Supplementary Figure 1.** Self-pairwise distances between no- and yes-glucosamine groups are not significantly different in a dataset lacking dogs Sami and Kesha. No-glucosamine to yes-glucosamine pairwise distances were calculated for each dog, excluding Sami and Kesha (n=22) and then a Wilcoxon rank-sum test was performed between distance metrics and glucosamine supplementation status (yes n=4, no n=18).


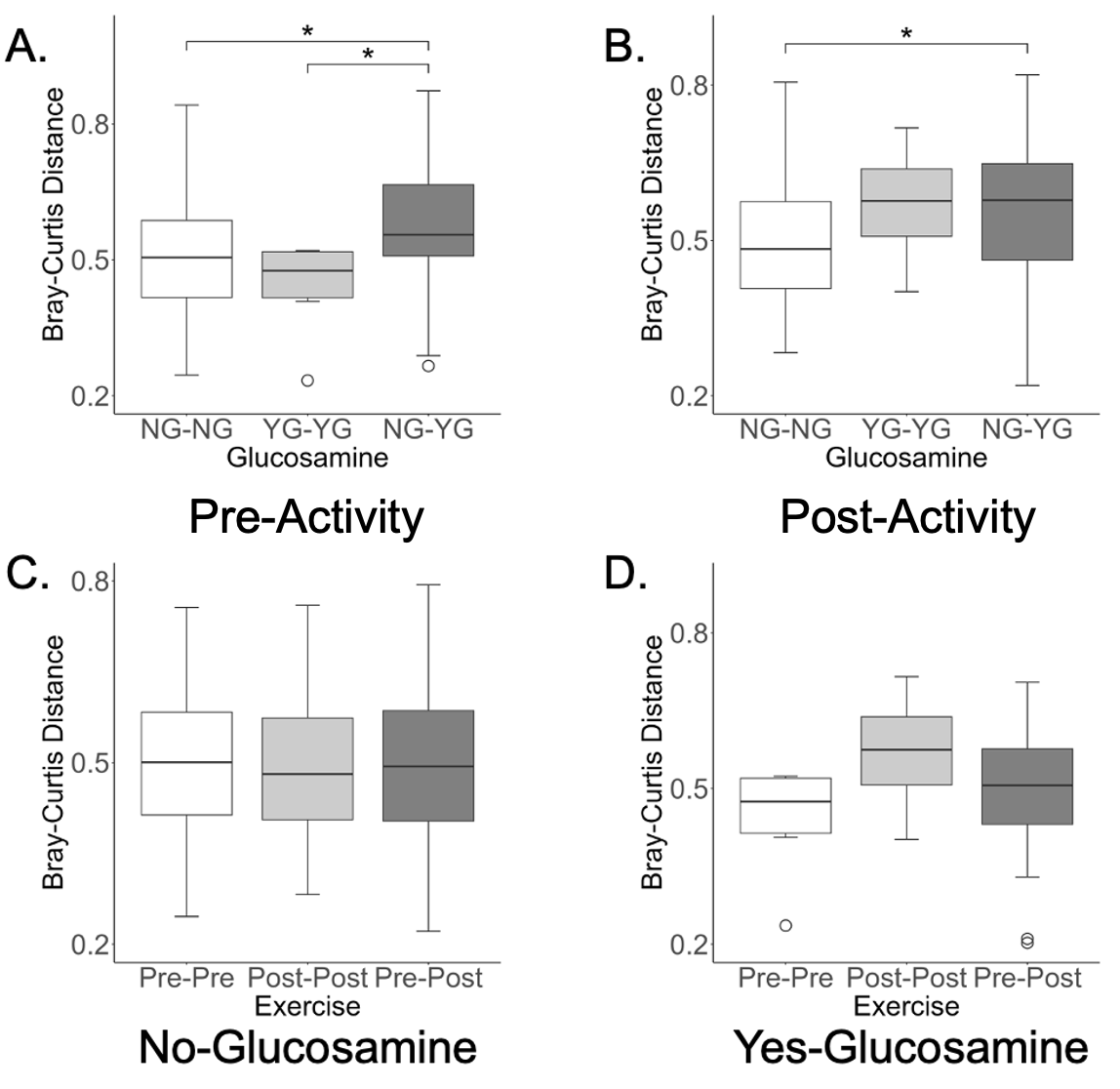


**Supplementary Figure 2.** Interindividual pairwise distance analysis reveals variation in beta-diversity distances in pre-activity, post-activity, and glucosamine supplementation groups in a dataset with dogs Sami and Kesha removed. Excluding Sami and Kesha, every pairwise Bray-Curtis beta-diversity distance was calculated in **(A)** pre-activity samples, **(B)** post-activity samples, **(C)** no glucosamine samples, and **(D)** glucosamine supplementation samples. († = p<0.10, * = p<0.05, ** = p<0.001 as determined by the Wilcoxon rank-sum test with Benjamini-Hochberg p-value correction).


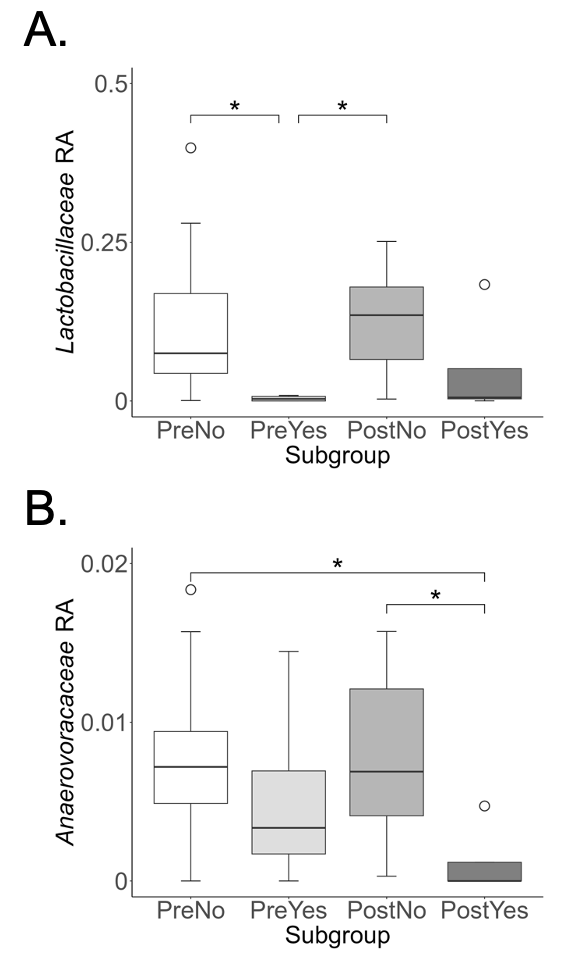


**Supplementary Figure 3.** *Lactobacillaceae* and *Anaerovoracaceae* taxa abundance plots reveal differences in relative abundance by population subgroups in a dataset with dogs Sami and Kesha removed. Relative abundance analysis was performed excluding Sami and Kesha for (**A**) *Lactobacillaceae* and (**B**) *Anaerovoracaceae* families for pre-activity no-glucosamine, pre-activity yes-glucosamine, post-exercise no-glucosamine, and post-activity and yes-glucosamine population subgroups. (* = p<0.05 as determined by the Wilcoxon rank-sum test with Benjamini-Hochberg p-value correction)


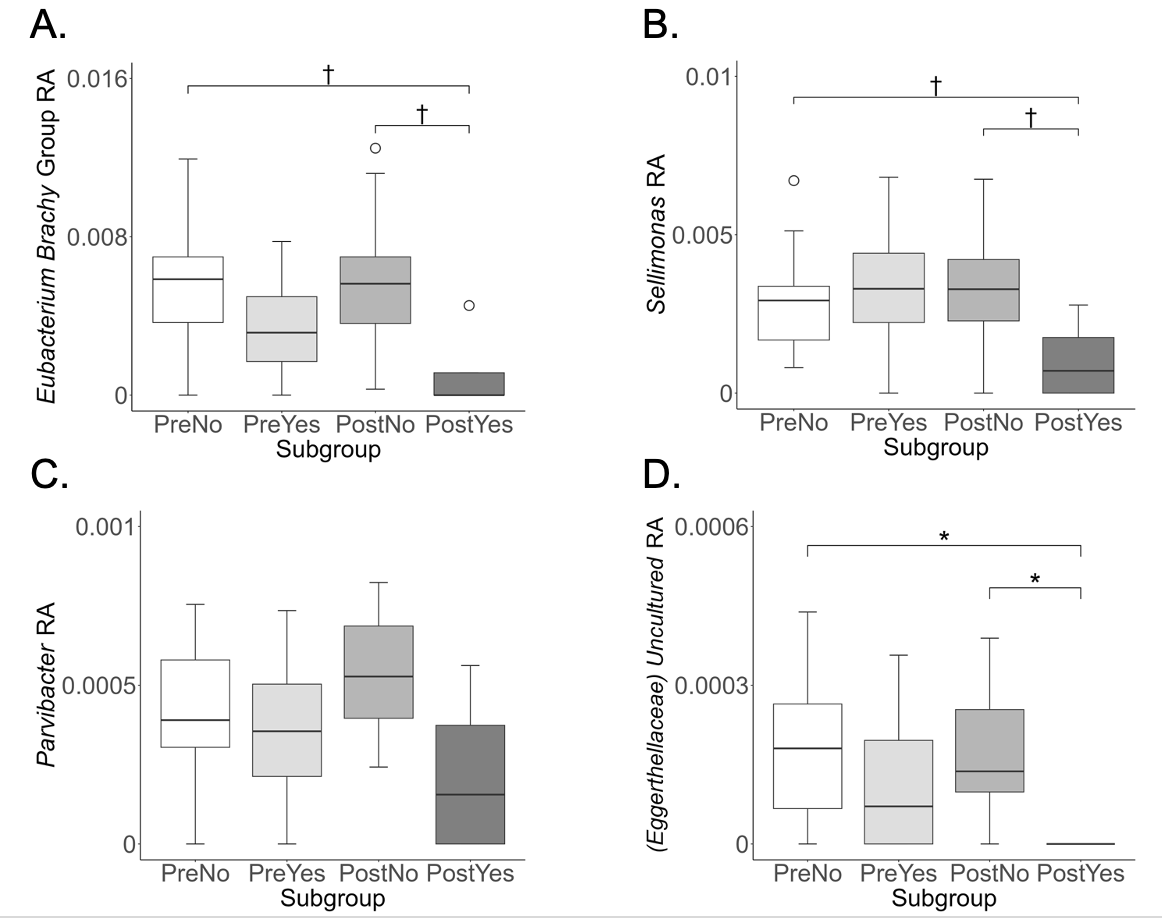


**Supplementary Figure 4.** *Eubacterium* [*brachy*], *Sellimonus*, *Parvibacter*, and uncultured genera taxa abundance plots reveal differences in relative abundance by population subgroups in a dataset not including dogs Sami or Kesha. Relative abundance analysis was performed in (**A**) *Eubacterium* [*brachy*] (**B**) *Sellimonas* (**C**) *Parvibacter*, and (**D**) Uncultured genera from the same family and *Parvibacter* for activity and glucosamine supplementation subgroups. (* = p<0.05, † = p<0.10 as determined by the Wilcoxon rank-sum test with Benjamini-Hochberg p-value correction)
